# Cystathionine *β*-Synthase Regulates the Proliferation, Migration, and Invasion of Thyroid Carcinoma Cells

**DOI:** 10.1155/2022/8678363

**Published:** 2022-06-27

**Authors:** Qi-Ying Jiang, Jian-Mei Li, Mi-Rong Jing, Yan-Xia Zhang, Qian-Qian Zhang, Chun-Bo Cai, Di Wang, Hui-Wen Qi, Tao Li, Yan-Zhang Li, Xin-Ying Ji, Dong-Dong Wu

**Affiliations:** ^1^School of Basic Medical Sciences, Henan University, Kaifeng, Henan 475004, China; ^2^Henan International Joint Laboratory for Nuclear Protein Regulation, Henan University, Kaifeng, Henan 475004, China; ^3^Kaifeng Municipal Key Laboratory of Cell Signal Transduction, Henan Provincial Engineering Centre for Tumor Molecular Medicine, Henan University, Kaifeng, Henan 475004, China; ^4^School of Stomatology, Henan University, Kaifeng, Henan 475004, China

## Abstract

Thyroid cancer is considered to be one of the most common endocrine tumors worldwide. Cystathionine *β*-synthase (CBS) plays a crucial role in the occurrence of several types of malignancies. And yet, the mechanism of action of CBS in the growth of thyroid carcinoma cells is still unrevealed. We found that CBS level in thyroid carcinoma tissue was higher than that in adjacent normal tissue. The overexpression of CBS enhanced the proliferation, migration, and invasion of thyroid cancer cells, while the downregulation of CBS exerted reverse effects. CBS overexpression reduced the levels of cleaved caspase-3 and cleaved poly ADP-ribose polymerase in thyroid cancer cells, whereas CBS knockdown showed reverse trends. CBS overexpression decreased reactive oxygen species (ROS) levels but increased the levels of Wnt3a and phosphorylations of phosphatidylinositol 3-kinase (PI3K), protein kinase B (PKB/AKT), mammalian target of rapamycin (mTOR), *β*-catenin, and glycogen synthase kinase-3 beta, while CBS knockdown exerted opposite effects. In addition, CBS overexpression promoted the growth of xenografted thyroid carcinoma, whereas CBS knockdown decreased the tumor growth by modulating angiogenesis, cell cycle, and apoptosis. Furthermore, aminooxyacetic acid (an inhibitor of CBS) dose-dependently inhibited thyroid carcinoma cell growth. CBS can regulate the proliferation, migration, and invasion of human thyroid cancer cells via ROS-mediated PI3K/AKT/mTOR and Wnt/*β*-catenin pathways. CBS can be a potential biomarker for diagnosing or prognosing thyroid carcinoma. Novel donors that inhibit the expression of CBS can be developed in the treatment of thyroid carcinoma.

## 1. Introduction

Hydrogen sulfide (H_2_S) has been recognized as an important gasotransmitter following nitric oxide and carbon monoxide [1–3]. H_2_S is generated from L-cysteine (L-Cys) mainly regulated by two pyridoxal-5′-phosphate-dependent enzymes, namely, cystathionine *γ*-lyase (CSE) and cystathionine *β*-synthase (CBS). CSE and CBS are predominantly localized to the cytoplasm [3–5]. With the occurrence of *α*-ketoglutarate, 3-mercaptopyruvate sulfurtransferase (3-MST) can work along with cysteine aminotransferase (CAT) to generate H_2_S from L-Cys. Both 3-MST and CAT have been found in mitochondria and cytoplasm [5, 6]. In addition, D-amino acid oxidase can convert D-cysteine to 3-mercaptopyruvate, that is, a substrate for 3-MST to generate H_2_S in brain and kidney [7]. H_2_S could be scavenged by methaemoglobin or by disulfide- or metallo-containing molecules that act as bound-sulfate and sulfane-sulfur pools. Methylation and oxidation are other pathways for H_2_S metabolism [3].

Under physiological conditions, H_2_S plays important roles in angiogenesis [8], energy production [9], neuronal activity [10], glucose regulation [11], and vascular relaxation [12]. Nevertheless, aberrant H_2_S metabolism has been noticed in several diseases, such as hypertension [13], asthma [14], atherosclerosis [15], cancer [16], diabetes [17], and neurodegenerative diseases [18]. Thyroid carcinoma is considered one of the most prevalent endocrine malignancies with a dramatic global increase in incidence during recent few years [19]. We have demonstrated that treatment with exogenous H_2_S can regulate the growth of human thyroid cancer [20]. In addition, it has been demonstrated that CBS level is increased in thyroid carcinoma compared to benign thyroid tissue [21], suggesting that CBS may be linked to the development of thyroid cancer. Nevertheless, the mechanism of action of CBS in the growth of human thyroid cancer remains unrevealed.

In the current study, we determined the mechanism of action of CBS in the proliferation, migration, invasion, and cell cycle progression of human thyroid cancer and investigated the effect of CBS on the growth of xenografted thyroid carcinoma. The role of aminooxyacetic acid (AOAA, an inhibitor of CBS) in thyroid carcinoma cell growth was further detected.

## 2. Materials and Methods

### 2.1. Materials/Animals

Human thyroid carcinoma specimens and corresponding adjacent normal tissues were purchased from National Human Genetic Resources Sharing Service Platform (Shanghai, China). Fresh specimens were collected from patients who had undergone surgery. Human thyroid carcinoma cell lines (ARO, TT, TPC-1, and FTC-133) and normal human thyroid epithelial cell line Nthy-ori3-1 were obtained from Cobioer Biosciences (Nanjing, Jiangsu, China). RPMI 1640 medium, fetal bovine serum (FBS), penicillin, streptomycin, and AOAA were purchased from Sigma-Aldrich (St. Louis, MO, USA). GV230 and GV102 were purchased from Genechem (Shanghai, China). Lipofectamine 3000 reagent was purchased from Thermo Fisher Scientific (Carlsbad, CA, USA). G418 and the RIPA lysis buffer were purchased from Solarbio (Beijing, China). Apollo 567 *in vitro* imaging kit was purchased from RiboBio (Guangzhou, Guangdong, China). CellTiter 96 AQ_ueous_ one solution cell proliferation assay kit was purchased from Promega (Madison, WI, USA). Total superoxide dismutase (SOD, Cat#S0101), catalase (CAT, Cat#S0051), and glutathione peroxidase (GSH-Px, Cat#S0056) detection kits, as well as *in situ* cell death detection kit were purchased from Beyotime (Haimen, Jiangsu, China). Reactive oxygen species (ROS) detection assay kit was obtained from Applygen Technologies Inc. (Cat#C1300, Beijing, China). Anti-CSE, anti-CBS, anti-3-MST, anti-Cyclin D1, anti-Cyclin E1, anti-cyclin-dependent kinase (CDK)2, anti-CDK4, anti-p21, anti-p27, antiphosphatidylinositol 3-kinase (PI3K), anti-phospho (p)-PI3K (Tyr199/Tyr458), antiprotein kinase B (PKB/AKT), anti-p-AKT (Ser473), antimammalian target of rapamycin (mTOR), anti-p-mTOR (Ser2448), anti-*β*-catenin, anti-p-*β*-catenin (Ser552), anti-Wnt3a, antiglycogen synthase kinase-3 beta (Gsk-3*β*), anti-p-Gsk-3*β* (Ser9), anti-CD31, and anti-Ki67 antibodies were obtained from Cell Signaling Technology (CST, Danvers, MA, USA). Anti-B-cell lymphoma-extra large (Bcl-xl), anti-B-cell lymphoma-2 (Bcl-2), anti-Bcl-xl/Bcl-2-associated death promoter (Bad), anti-Bcl-2-associated X protein (Bax), anti-Cleaved caspase-3, anti-Cleaved poly-ADP-ribose polymerase (PARP), anti-*β*-actin antibodies, and the horseradish peroxidase-conjugated secondary antibody were obtained from Proteintech (Chicago, IL, USA). H_2_S detection assay kit was purchased from LanpaiBio (Cat#hj-C2452, Shanghai, China). BALB/C nude mice were obtained from Vital River Laboratory Animal Technology Co., Ltd. (Beijing, China).

### 2.2. Tissue Samples

The protein level of CBS was determined in 54 human thyroid carcinoma specimens and corresponding adjacent normal tissues by using immunohistochemistry (IHC). The clinical study was approved by the Ethics Committee of the First Affiliated Hospital of Henan University (20200212), and the written informed consent was obtained from each subject. Then 4 fresh specimens were used to detect the expression level of CBS. Clinicopathological staging and histological classification were specified according to the American Joint Committee on Cancer criteria [22].

### 2.3. Evaluation of Immunohistochemical Staining

The immunohistochemical staining was reported in an independent manner by two separate experienced pathologists. The immunohistochemical results were scored in a semiquantitative manner based on the percentage of stained cells (0, 0%; 1, 1-25%; 2, 26-50%; 3, 51-75%; and 4, 76-100%) and the intensity of that staining (0, negative; 1, weak; 2, moderate; and 3, strong). Then, the two percentages were put together to calculate one final score of CBS. The combined scores were categorized as 0-3, low expression and 4-7, high expression [23].

### 2.4. Cell Culture

Cells were cultured in RPMI 1640 medium supplemented with 10% FBS, 100 units/ml penicillin, and 100 *μ*g/ml streptomycin in a humidified atmosphere with 95% air/5% CO_2_ at 37°C. ARO and TPC-1 cells were, respectively, treated with 2.5, 5, 10, and 20 mM AOAA for 24 h. The control group was treated with PBS for 24 h.

### 2.5. Overexpression and Knockdown of CBS

Human CBS complementary deoxyribonucleic acid (cDNA) (NM_000071) was subcloned between the EcoRI and BamHI sites of GV230, identified by gene sequencing, and transfected into human thyroid cancer cells using Lipofectamine 3000 reagent. The empty vector (Mock group) and GV230-CBS construct (CBS group) were, respectively, transfected into cancer cells. The oligonucleotides encoding short hairpin ribonucleic acid (shRNA) specific for the scramble sequences and CBS were cloned into the BamHI and Hind III sites of GV102, respectively. The scramble shRNA (sh-Scb group) and CBS shRNA (sh-CBS group) were verified by gene sequencing and transfected into cancer cells. Then, G418 was used to screen the stable cell lines. The untransfected cells were used as a negative control group. 72 hours after transfection was performed, the localization of CBS was observed using fluorescent microscopy (Eclipse Ti, Nikon, Melville, NY, USA).

### 2.6. Cell Proliferation and Viability Assays

Cell proliferation was detected by the 5-ethynyl-2′-deoxyuridine (EdU) staining assay using the Apollo 567 *in vitro* imaging kit. Cells were visualized using a fluorescent microscopy. The rate of cell proliferation was measured as the percentage of EdU-positive cells to total cells [24]. Cell viability was detected utilizing the CellTiter 96 AQ_ueous_ one solution cell proliferation assay kit.

### 2.7. Colony Formation Assay

Cells were cultured in six-well plates with a seeding number of 8 × 10^2^ cells in each well at 37°C for 2 weeks. The colonies were fixed using methanol for 10 min, and then, staining was performed using 0.5% crystal violet at room temperature for 30 min. The plates were photographed, and the number of colonies in each well was reported [25].

### 2.8. Flow Cytometry

Cells were detached using trypsin, washed using phosphate-buffered saline (PBS), and finally fixed using ice-cold 75% ethanol. Then, the cells were washed again with PBS and incubated with propidium iodide (PI) and RNase A for 30 min at room temperature. Cell cycle was detected using a BD FACSVerse flow cytometer (San Jose, CA, USA).

### 2.9. Wound Healing Assay

Cells were cultured as monolayer then wounded by a sterile tip of a 200 *μ*l pipette. A CKX41 inverted microscope (Olympus, Tokyo, Japan) was used to observe cell migration. Images were then analyzed by Image J software (NIH, Bethesda, MD, USA). The migration rate (MR) was measured using the formula: MR = [(*A* − *B*)/*A*] × 100%, where *A* and *B* represent the width at 0 h and 24 h, respectively [26].

### 2.10. Soft Agar Assay

A cell suspension was formed using a medium containing 10% FBS and 0.6% agarose. Then, the mixture was added to a lower layer formed of 1.2% agarose that was set in six-well plates with the density of 1 × 10^4^ cells/well. The medium was renewed every other 3 days. The colonies were observed after 2 weeks with an Olympus CKX41 inverted microscope, and the number of colonies in each well was reported [25].

### 2.11. Migration and Invasion Assays

Transwell migration and invasion assays were done the same way as previously mentioned [27]. The number of positively stained cells was analyzed by a Zeiss Axioskop 2 plus microscope (Thornwood, NY, USA).

### 2.12. TdT-Mediated dUTP-Biotin Nick End Labeling (TUNEL) Assay

TUNEL staining method was carried out using the *in situ* cell death detection kit. The apoptotic cells were reported using fluorescent microscopy (Eclipse Ti). The percentage of TUNEL-positive cells was analyzed by using Image J software.

### 2.13. Detection of ROS

The levels of ROS within the cells were determined by the dihydroethidium (DHE) cellular ROS detection assay kit. The cells were washed with PBS and incubated with 10 *μ*M DHE in fresh serum-free media at 37°C for 30 min in the dark. After washing, images were obtained with fluorescent microscopy (Eclipse Ti), and the fluorescence intensity was quantified with Image J software.

### 2.14. Measurements of Antioxidant Activities

Cells were cultured in 6-well plates (1 × 10^5^ cells/well) for 24 h. Then, the RIPA lysis buffer was added, and the supernatant was collected to obtain the total protein. Subsequently, the activities of total SOD, CAT, and GSH-Px were detected using commercial kits according to the instructions.

### 2.15. Western Blot

Western blot was conducted to detect the protein expression levels of relevant proteins as previously described [26]. The immunoreactive protein bands were depicted using a chemiluminescence machine (Thermo, Rockford, IL, USA).

### 2.16. Animal Study

Animal experiments and care were approved by the Committee of Medical Ethics and Welfare for Experimental Animals of Henan University School of Medicine (HUSOM-2020-022) in accordance with the guidelines of experimental animal regulations formulated by the National Science and Technology Commission, China. Animal study was conducted according to the formerly mentioned method with minor alterations [27]. BALB/C nude mice (4-week-old, male) were divided as 6 mice per group. TPC-1 and ARO cells were harvested and washed with PBS. Then, the cells were pelleted by brief centrifugation at 300 × g. The supernatant was removed, and the cells were resuspended in PBS with a density of 1 × 10^7^ cells/ml. 2 × 10^6^ cells (in 200 *μ*l PBS) with overexpression or knockdown of CBS were subcutaneously injected into the right flank area of nude mice. For the CBS inhibitor experiment, AOAA (2.5, 5, 10, and 20 mg/kg/day) was injected subcutaneously for 4 weeks. The control group was injected with PBS for 4 weeks. The tumor volumes and body weights of nude mice were daily monitored. The tumor volume was estimated according to the following formula: volume (*V*) = *L* × *W*^2^/2, where *L* represents the longest dimension parallel to the skin surface, and *W* represents the dimension perpendicular to *L* [20]. The parameter of DT/DC (%) was estimated, where DT = *T* − Do and DC = *C* − Do (*T*/*C* represents the volumes of the treated/untreated tumors; Do represents the average tumor volume at the start of the experiment) [28]. The tumor volume doubling time (TVDT) was estimated as TVDT = log2/log(*V*2/*V*1) × (*T*–*T*_0_), where *V*2/*V*1, respectively, represents the volume of tumors at two measurement times and (*T*–*T*_0_) represents the time period [23]. The tumors were removed and weighted after performing the experiment. The tumor growth inhibition rate (IR) = [(*A* − *B*)/*A*] × 100%, where *A* represents the average tumor weight of the control group, and *B* represents that of the treatment group [25].

### 2.17. Measurements of H_2_S Concentrations

The levels of H_2_S in cells, culture supernatant, and tissues were determined by the enzyme-linked immunosorbent assay kit. Briefly, 50 *μ*L of streptavidin-horseradish peroxidase (HRP) and 50 *μ*L of standard solution were added to the wells in the antibody precoated microplates. Then, 40 *μ*L of samples to be tested, 50 *μ*L of streptavidin-HRP, and 10 *μ*L of H_2_S-antibody were added to each well. The plates were covered and incubated at 37°C for 60 min. After incubation, the wells were decanted and washed 5 times with the 20 × wash solution. Then, the wells were incubated with 50 *μ*L chromogen A and 50 *μ*L chromogen B at 37°C for 15 min in dark. To stop the reaction, 50 *μ*L of the stop solution was added to each well which immediately turned the color of the solution into yellow. For the blank wells, only chromogen A, chromogen B, and the stop solution were added. The optical density (OD) of each well was determined spectrophotometrically at 450 nm using a SpectraMax M2 microplate reader (Molecular Devices, Sunnyvale, CA, USA). The value for the blank was subtracted from both the samples and standard controls. A calibration curve was plotted relating the concentration of each standard solution on the *X* axis to the OD value on the *Y* axis. The standard curve linear regression equation was created, and the H_2_S concentration was calculated from the standard curve.

### 2.18. Hematoxylin and Eosin (HE) Staining

Tumor tissues were fixed using 10% neutral buffered formalin, embedded in paraffin, sectioned at 5 *μ*m thickness, and performed according to HE staining protocol [27]. The results were depicted under a Zeiss Axioskop 2 plus microscope.

### 2.19. IHC

Cluster of differentiation 31 (CD31) is considered a crucial biomarker for vascular endothelial cells. The staining density of CD31 has been considered as the tumor microvessel density (MVD) [29]. Tumor tissues were stained using anti-CD31, anti-Ki67, anti-p21, and anti-cleaved caspase-3 antibodies, respectively. The results were depicted under a Zeiss Axioskop 2 plus microscope. Then, the MVD was calculated, and the proliferation index, p21 positive cells, and apoptotic index were determined by the ratios of the positively stained cells to the total number.

### 2.20. Statistical Analysis

All the obtained data are expressed as mean and standard error of the mean (SEM). Differences among different groups were interpreted using one-way analysis of variance by the software SPSS followed by Tukey's test. *P* < 0.05 was considered statistically significant.

## 3. Results

### 3.1. CBS Is Upregulated in Human Thyroid Carcinoma Cell Lines and Tissues

**Figure 1:**
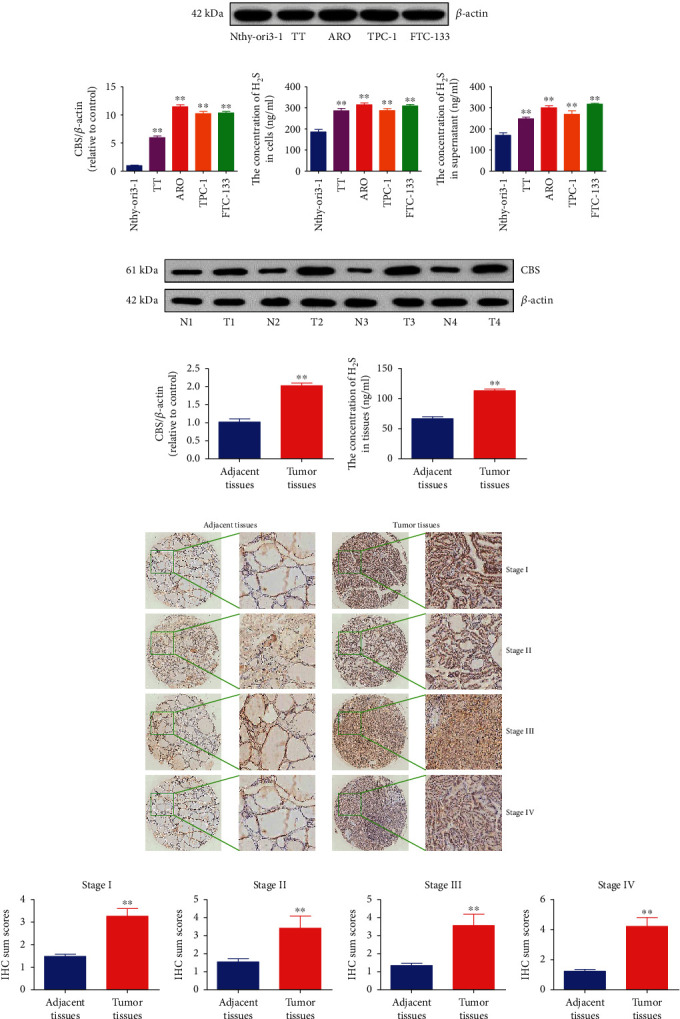
The expression levels of CBS in human thyroid carcinoma cell lines and tissues. (a) Western blotting analysis for the expression levels of CBS in Nthy-ori3-1, TT, ARO, TPC-1, and FTC-133 cells. *β*-Actin was used as the loading control. (b) The densitometry analyses of CBS were performed, normalized to the corresponding *β*-actin level. (c, d) The concentrations of H_2_S in cells and culture supernatant were determined. Data are presented as mean ± SEM of three independent experiments; ∗∗*P* < 0.01 compared with human thyroid epithelial cell line Nthy-ori3-1. (e) The expression levels of CBS in fresh human thyroid carcinoma tissues (T) and adjacent normal tissues (N) were detected by Western blotting. *β*-Actin was used as the loading control. (f) The densitometry analyses of CBS were performed, normalized to the corresponding *β*-actin level. (g) The concentrations of H_2_S in human thyroid carcinoma tissues and adjacent normal tissues were determined. (h) IHC results of CBS expression in different clinical stages of human thyroid carcinoma tissues and adjacent tissues (left: 400 ×; right: enlarged). (i) IHC sum scores were adopted to compare CBS expression in different clinical stages of human thyroid carcinoma tissues and adjacent tissues. Data are presented as mean ± SEM of three independent experiments; ∗∗*P* < 0.01 compared with adjacent normal tissues.

We firstly determined the level of CBS in human thyroid carcinoma cell lines. The protein expressions of CBS were dramatically increased in all human thyroid cancer cell lines compared with normal thyroid cell line (Figures 1(a) and 1(b)). While the levels of CSE and 3-MST were different between human thyroid carcinoma cell lines and normal thyroid cell line (Figure S1). In addition, the concentrations of H_2_S in human thyroid carcinoma cells and supernatant were found to be higher than those in normal thyroid cells and supernatant (Figures 1(c) and 1(d)). We further determined the protein levels of CBS in fresh tissues of thyroid cancer and their surrounding nontumor tissues. In agreement with the above findings, the expression levels of CBS were high in thyroid carcinoma tissues and low in adjacent normal tissues (Figures 1(e) and 1(f)). H_2_S levels in thyroid carcinoma tissues were higher than those in adjacent normal tissues (Figure 1(g)). Furthermore, CBS expression level in human thyroid carcinoma was detected using a tissue chip that consists of 54 thyroid carcinoma samples and surrounding tissues using IHC. The data indicated that CBS expression was higher in each clinical stage of human thyroid carcinoma compared with adjacent tissues (Figures 1(h) and 1(i)). To study the clinical impact of CBS in human thyroid carcinoma, the association of CBS levels to clinicopathological parameters in thyroid carcinoma tissue chip was further analyzed (Table 1). CBS expression was associated with T classification of thyroid carcinoma. The data suggest that CBS can be a potential marker for the diagnosis and prognosis of thyroid carcinoma. In addition, it can be a growth regulator in thyroid carcinoma cells.

### 3.2. CBS Mediates the Proliferation and Viability of Human Thyroid Carcinoma Cells

**Figure 2:**
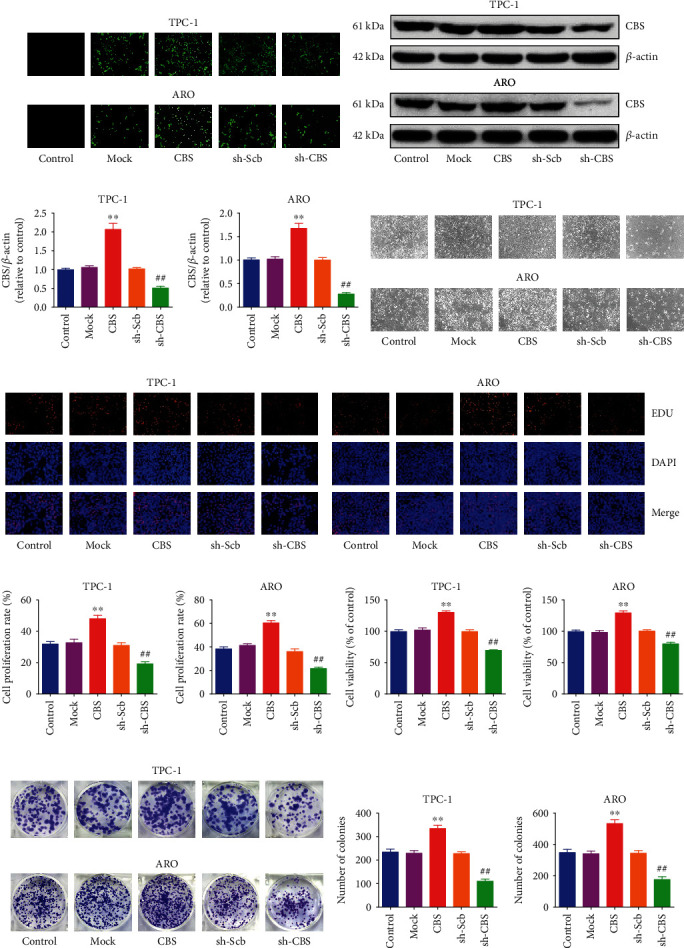
Effects of CBS on the proliferation and viability of human thyroid carcinoma cells. (a) Fluorescence microscopy of CBS in TPC-1 and ARO cells; original magnification 100×. (b) The protein expression of CBS was examined by Western blotting. *β*-Actin was used as the loading control. (c) The densitometry analysis of CBS was performed, normalized to the corresponding *β*-actin level. (d) Phase-contrast microscopy of CBS in TPC-1 and ARO cells; original magnification 100×. (e) DNA replication activities of TPC-1 and ARO cells in each group were examined by EdU assay; original magnification 200×. (f) The proliferation rate of each group was analyzed. (g) The percentages of viable cells were determined using MTS, and the cell viability of the control group was taken as 100%. (h) The clonogenic capacity was determined in TPC-1 and ARO cells. (i) The numbers of colonies were calculated. Data are presented as mean ± SEM of three independent experiments; ∗∗*P* < 0.01 compared with the Mock group; ^##^*P* < 0.01 compared with the sh-Scb group.

To investigate the impact of CBS on the growth of human thyroid carcinoma cells, CBS knockdown and overexpression cells were constructed, and experiments were conducted to compare different groups. Transfection of CBS cDNA into TPC-1 and ARO cells enhanced the protein levels of CBS and transfection of sh-CBS caused less expression of CBS in both TPC-1 and ARO cells (Figure 2(a)). The protein expression levels of CBS showed similar results (Figures 2(b) and 2(c)). Furthermore, CBS overexpression significantly enhanced the levels of H_2_S in human thyroid carcinoma cells and supernatant, whereas CBS knockdown exhibited reverse trends (Figure S2). These results suggest successful experiments of CBS overexpression and knockdown. As shown in Figures 2(d)–2(f), CBS overexpression enhanced the proliferation of TPC-1 and ARO cells compared with the Mock group, while CBS knockdown exhibited reverse effects compared with the sh-Scb group. CBS exerted similar effects when experimenting the viability of human thyroid carcinoma cells (Figure 2(g)). Moreover, CBS overexpression increased the number of colonies, and CBS knockdown significantly decreased it (Figures 2(h) and 2(i)). Taken together, the above data indicate that CBS can be involved in the proliferation and viability of human thyroid carcinoma cells.

### 3.3. CBS Regulates Cell Cycle Progression in Human Thyroid Carcinoma Cells

**Figure 3:**
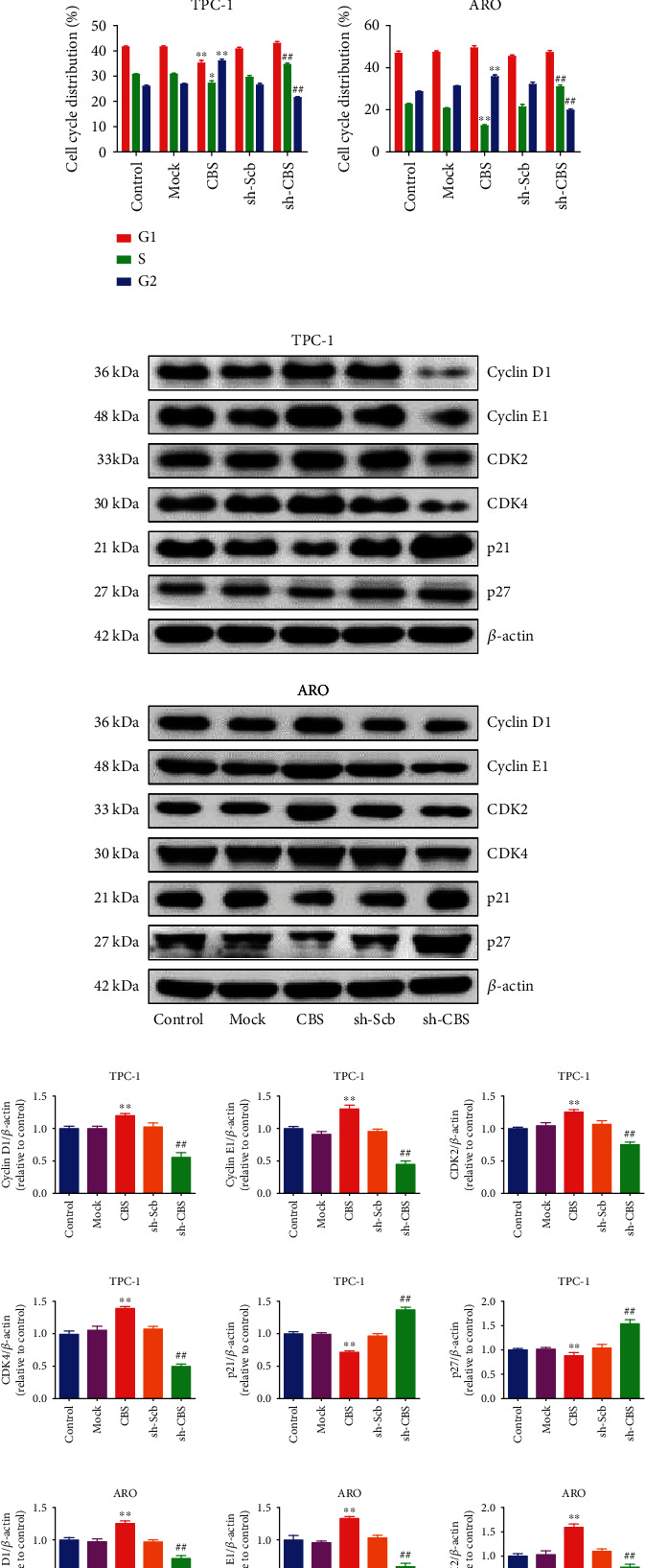
Effects of CBS on cell cycle progression of human thyroid carcinoma cells. (a) Flow cytometry assay was used to determine cell cycle distribution. (b) Cell cycle distribution was analyzed. (c) Western blot analysis for the expression levels of Cyclin D1, Cyclin E1, CDK2, CDK4, p21, and p27 in each group. *β*-Actin was used as the loading control. (d) The densitometry analysis of each factor was performed, normalized to the corresponding *β*-actin level. Data are presented as mean ± SEM of three independent experiments; ∗*P* < 0.05, ∗∗*P* < 0.01 compared with the control group; ^##^*P* < 0.01 compared with the OA group.

In order to determine whether CBS could affect the cell cycle progression, flow cytometry was conducted. CBS overexpression caused increased percentage of cell population in G2 phase and induced a decrease in the percentage of cells in S phase, whereas CBS knockdown exhibited reverse trends (Figures 3(a) and 3(b)). Many cell cycle-related proteins are involved in regulating cell cycle progression, such as CDK2, CDK4, cyclin D1, cyclin E1, p21, and p27 [30, 31]. As shown in Figures 3(c) and 3(d), CBS overexpression caused an increase in the expressions of cyclin D1, cyclin E1, CDK2, and CDK4, but resulted in a decrease in the levels of p21 and p27. However, CBS knockdown exerted reverse effects on the expressions of these proteins. The results together conclude that CBS can regulate cell cycle in human thyroid carcinoma cells.

### 3.4. CBS Mediates the Migration and Invasion of Human Thyroid Carcinoma Cells

**Figure 4:**
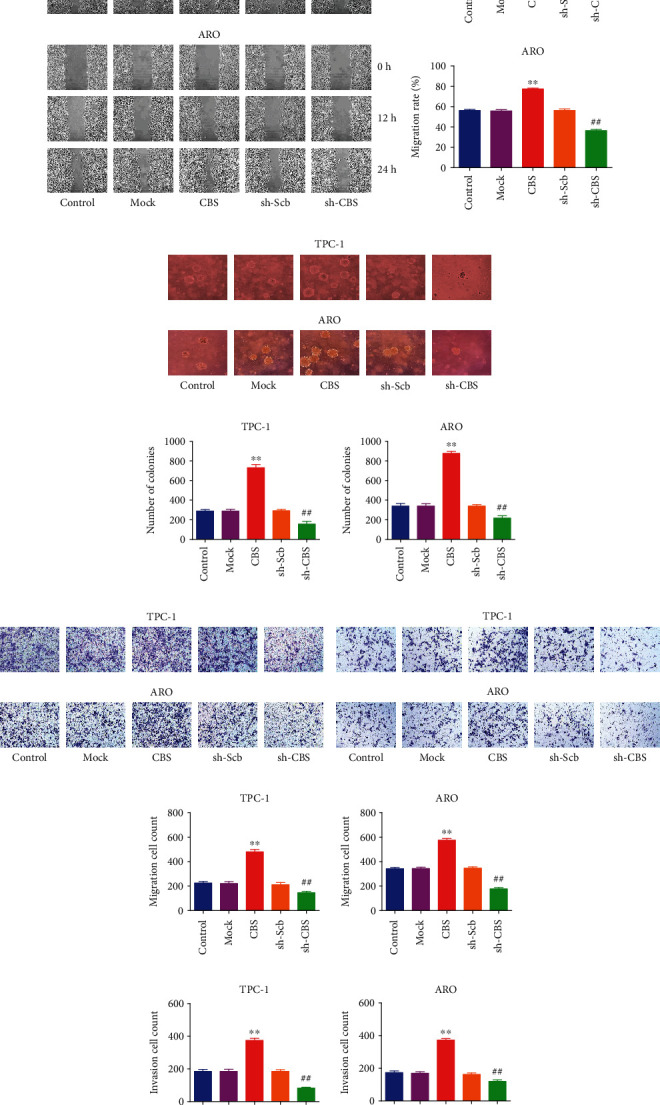
Effects of CBS on the migration and invasion of human thyroid carcinoma cells. (a) The effect of CBS on cell migration was measured by wound healing assay; original magnification 100×. (b) The migration rates of TPC-1 and ARO cells were calculated. (c) Soft agar assay was performed to examine the anchorage-independent survival of cells; original magnification 100×. (d) The number of colonies was calculated. (e) Transwell assay was performed to assess the migration of TPC-1 and ARO cells; original magnification 200×. (f) Transwell assay was performed to assess the invasion of TPC-1 and ARO cells; original magnification 200×. (g) The numbers of the migrated cells were calculated. (h) The numbers of the invasive cells were calculated. Data are presented as mean ± SEM of three independent experiments; ∗∗*P* < 0.01 compared with the Mock group; ^##^*P* < 0.01 compared with the sh-Scb group.

A further evaluation of the effects of CBS on the migration and invasion of human thyroid carcinoma cells was conducted. As for the scratch test to assess migration, CBS overexpression increased the migration capacity of TPC-1 and ARO cells, and CBS knockdown demonstrated opposite effects (Figures 4(a) and 4(b)). As for soft agar assay, overexpression of CBS promoted the anchorage-independent growth of TPC-1 and ARO cells, while opposite trends were observed in sh-CBS group (Figures 4(c) and 4(d)). As for the Transwell analysis, results indicated that the migration and invasion capabilities of TPC-1 and ARO cells were increased in CBS group, whereas the sh-CBS group showed reverse effects (Figures 4(e)–4(h)). The data suggest that CBS can affect the migration and invasion of human thyroid carcinoma cells.

### 3.5. CBS Modulates Mitochondrial Apoptosis in Human Thyroid Carcinoma Cells

**Figure 5:**
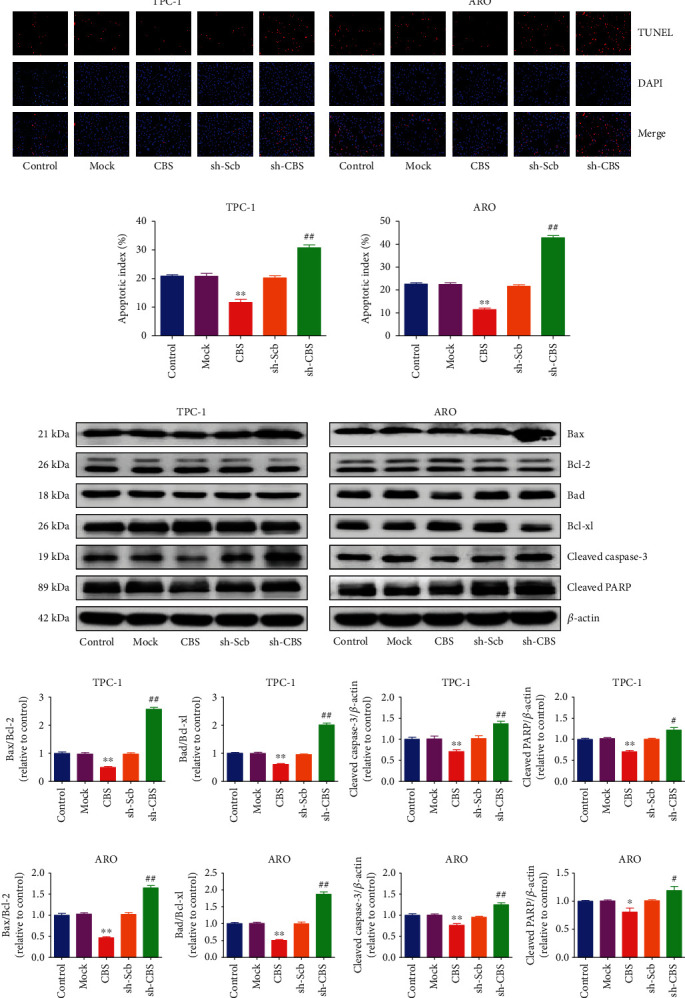
Effects of CBS on the apoptosis in human thyroid carcinoma cells. (a) The apoptotic levels of TPC-1 and ARO cells were measured by TUNEL staining; original magnification 100×. (b) The percentages of TUNEL-positive cells were calculated. (c) Western blotting analysis for the expression levels of Bax, Bcl-2, Bad, Bcl-xl, cleaved caspase-3, and cleaved PARP in TPC-1 and ARO cells. *β*-Actin was used as the loading control. (d) The densitometry analysis of each factor was performed, normalized to the corresponding *β*-actin level. The expression ratios of Bax/Bcl-2 and Bad/Bcl-xl were quantified. Data are presented as mean ± SEM of three independent experiments; ∗*P* < 0.05, ∗∗*P* < 0.01 compared with the Mock group; ^#^*P* < 0.05, ^##^*P* < 0.01 compared with the sh-Scb group.

Figures 5(a) and 5(b) showed that the apoptotic index decreased in the CBS group in contrast with the Mock group and increased in the sh-CBS group in contrast with the sh-Scb group. The Bcl-2 family proteins act as effector molecules in regulating mitochondrial apoptosis, including the proapoptotic proteins Bad and Bax, as well as the antiapoptotic proteins Bcl-xl and Bcl-2 [32]. Increased Bad/Bcl-xl and Bax/Bcl-2 ratios have been found in mitochondrial apoptosis [33, 34]. Caspase-3 is a caspase family member that induces cell apoptosis via mitochondria-mediated pathway [35]. PARP is involved in repairing damaged DNA and has been recognized as a substrate for caspase-3 cleavage during apoptosis [36]. Our results indicated that CBS overexpression downregulated the Bad/Bcl-xl and Bax/Bcl-2 ratios, as well as the expressions of cleaved caspase-3 and cleaved PARP, while CBS knockdown demonstrated reverse trends (Figures 5(c) and 5(d)). The results show that CBS can modulate mitochondria-dependent apoptosis in human thyroid carcinoma cells.

### 3.6. CBS Modulates ROS-Mediated PI3K/AKT/mTOR and Wnt/*β*-Catenin Signaling Pathways in Human Thyroid Carcinoma Cells

**Figure 6:**
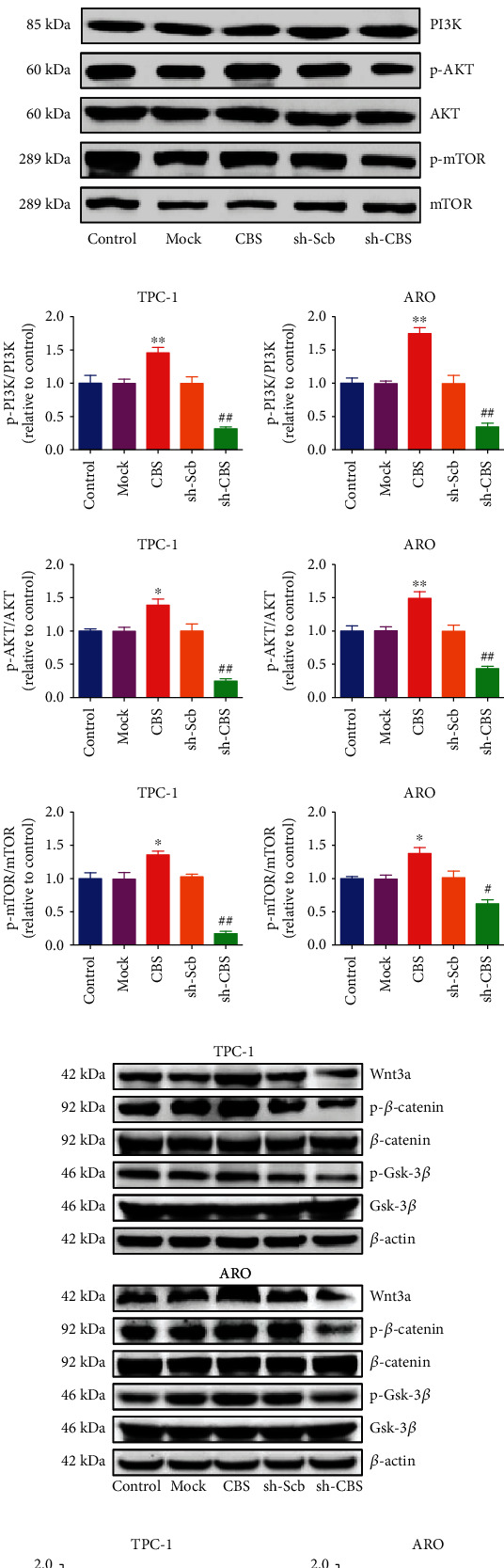
Effects of CBS on ROS-mediated PI3K/AKT/mTOR and Wnt/*β*-Catenin signaling pathways in human thyroid carcinoma cells. (a) The intracellular ROS production was detected using the fluorescent probes DHE (shown in red; original magnification, ×100). (b) The intracellular ROS production was measured (*n* = 6). (c) Western blotting analysis for the expression levels of p-PI3K, PI3K, p-Akt, Akt, p-mTOR, and mTOR in TPC-1 and ARO cells. *β*-Actin was used as the loading control. (d) The densitometry analyses of p-PI3K, PI3K, p-Akt, Akt, p-mTOR, and mTOR were performed in TPC-1 and ARO cells, normalized to the corresponding *β*-actin level. (e) Western blotting analysis for the expression levels of Wnt3a, p-*β*-catenin, *β*-catenin, p-Gsk-3*β*, and Gsk-3*β* in TPC-1 and ARO cells. *β*-Actin was used as the loading control. (f) The densitometry analyses of Wnt3a, p-*β*-catenin, *β*-catenin, p-Gsk-3*β*, and Gsk-3*β* were performed in TPC-1 and ARO cells, normalized to the corresponding *β*-actin level. Data are presented as mean ± SEM of three independent experiments; ∗*P* < 0.05, ∗∗*P* < 0.01 compared with the Mock group; ^#^*P* < 0.05, ^##^*P* < 0.01 compared with the sh-Scb group.

ROS are widely regarded as molecules containing oxygen with reactive properties [37, 38]. When ROS are at low or moderate levels, they can be involved in cellular signaling pathways as they can stimulate stress-responsive survival pathways and promote cell proliferation and differentiation. And yet, high levels of ROS induce injury of cellular components, including lipids, nucleic acids, and proteins [37, 39]. The results demonstrated that the overexpression of CBS can reduce ROS levels, and CBS knockdown increased the ROS levels in TPC-1 and ARO cells (Figures 6(a) and 6(b)). SOD, GSH-Px, and CAT enzymes play major roles in ROS-scavenging in human cells [40, 41]. CBS overexpression enhanced the activities of total SOD, CAT, and GSH-Px, while CBS knockdown showed reverse effects (Figure S3). ROS can serve in signaling processes that take place during various environmental stresses and play important roles in PI3K/AKT/mTOR and Wnt/*β*-Catenin pathways [42, 43]. The results showed that CBS overexpression upregulated the levels of p-PI3K, p-AKT, and p-mTOR in PI3K/AKT/mTOR pathway, as well as Wnt3a, p-*β*-catenin, and p-Gsk-3*β* in Wnt/*β*-Catenin pathway. However, CBS knockdown downregulated the expressions of these proteins (Figures 6(c)–6(f)). Overall, these data suggest that CBS modulates ROS-mediated PI3K/AKT/mTOR and Wnt/*β*-Catenin pathways in human thyroid carcinoma cells.

### 3.7. CBS Regulates the Growth of Human Thyroid Carcinoma Xenograft Tumors

**Figure 7:**
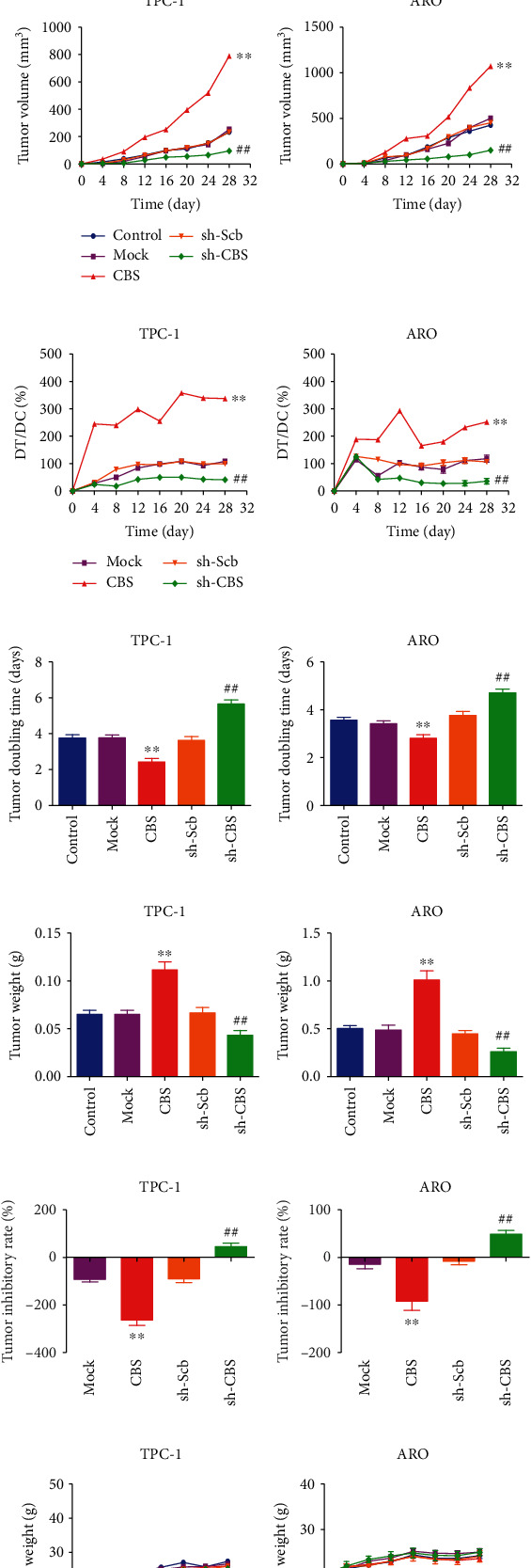
Effects of CBS on the growth of thyroid carcinoma xenograft tumors in nude mice. (a) Representative xenografts dissected from different groups of nude mice were shown. (b) Western blotting analysis for the expression levels of CBS in TPC-1 and ARO cells. *β*-Actin was used as the loading control. (c) The densitometry analysis of CBS was performed in TPC-1 and ARO cells, normalized to the corresponding *β*-actin level. (d)–(f) The tumor volume, DT/DC, and TVDT were calculated. (g, h) The tumors were weighed and the inhibition rates of tumor growth were calculated. (i, j) The body weight change curve of each group during the experiment and the body weight of each group on the first day (day 0) and the last day (day 28). Values are presented as mean ± SEM (*n* = 6); ∗∗*P* < 0.01 compared with the Mock group; ^##^*P* < 0.01 compared with the sh-Scb group.

TPC-1 and ARO cells are widely adopted to establish subcutaneous xenograft tumor models in nude mice [20, 36]. We then determined the effects of CBS on the growth of human thyroid carcinoma xenograft tumors. The tumors were removed and photographed at the end of the *in vivo* experiment (Figure 7(a)). The protein level of CBS in the tumor was further detected, the data indicated that the CBS level in CBS group was higher than that in Mock group, and the expression level of CBS in sh-CBS group was lower than that in sh-Scb group (Figures 7(b) and 7(c)). In addition, we observed that CBS overexpression dramatically promoted the growth of xenograft tumors, and CBS knockdown significantly decreased tumor growth (Figures 7(d)–7(h)). Furthermore, there was no significant difference in body weight among all groups (Figures 7(i) and 7(j)). IHC with Ki67 and CD31 antibodies suggested that the *in vivo* proliferation and MVD of thyroid carcinoma were increased in CBS group compared with the Mock group and decreased in the sh-CBS group compared with the sh-Scb group. Moreover, IHC with p21 and cleaved caspase-3 antibodies exhibited reverse trends (Figure S4). In sum, the results suggest that CBS can regulate the growth of xenografted human thyroid carcinoma.

### 3.8. CBS Inhibitor Suppresses Human Thyroid Carcinoma Cell Growth

**Figure 8:**
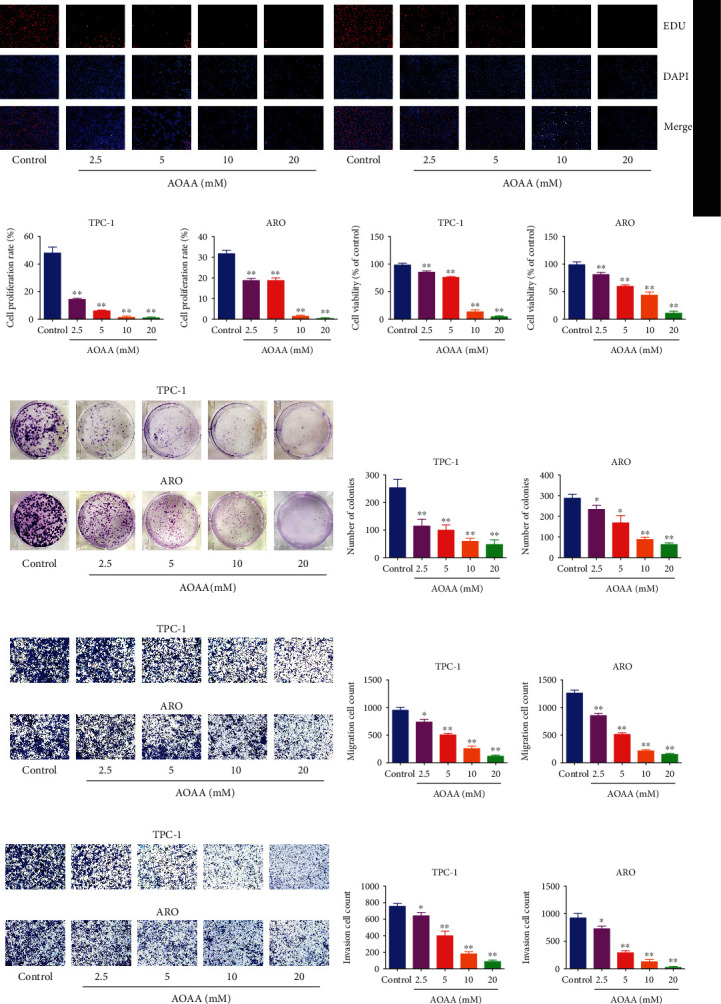
Effects of AOAA on the viability, proliferation, migration, and invasion of human thyroid carcinoma cells. (a) DNA replication activities of TPC-1 and ARO cells in each group were examined by EdU assay; original magnification 100×. (b) The proliferation rate of each group was analyzed. (c) The percentages of viable cells were determined using MTS and the cell viability of the control group was taken as 100%. (d) The clonogenic capacity was determined in TPC-1 and ARO cells. (e) The numbers of colonies were calculated. (f) Transwell assay was performed to assess the migration of TPC-1 and ARO cells; original magnification 200×. (g) The numbers of the migrated cells were calculated. (h) Transwell assay was performed to assess the invasion of TPC-1 and ARO cells; original magnification 200×. (i) The numbers of the invasive cells were calculated. Data are presented as mean ± SEM of three independent experiments; ∗*P* < 0.05, ∗∗*P* < 0.01 compared with the control group.

**Figure 9:**
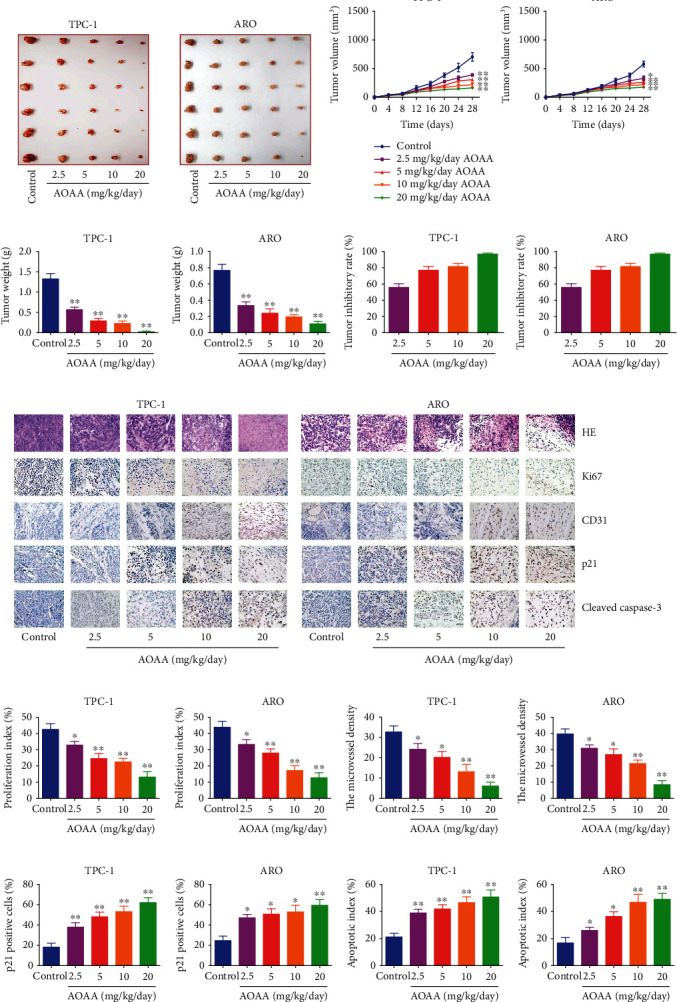
Effects of AOAA on thyroid carcinoma xenograft growth in nude mice. (a) Representative xenografts dissected from different groups of nude mice were shown. (b) The tumor volume was calculated. (c, d) The tumors were weighed and the inhibition rates of tumor growth were calculated. (e) Representative photographs of HE, Ki67, CD31, p21, and cleaved cas-3 staining in TPC-1 and ARO xenograft tumors (original magnification 400×). (f)–(i) The proliferation index, MVD, p21 positive cells, and apoptotic index were calculated. Data are presented as mean ± SEM of three independent experiments. ∗*P* < 0.05, ∗∗*P* < 0.01 compared with the control group.

**Figure 10:**
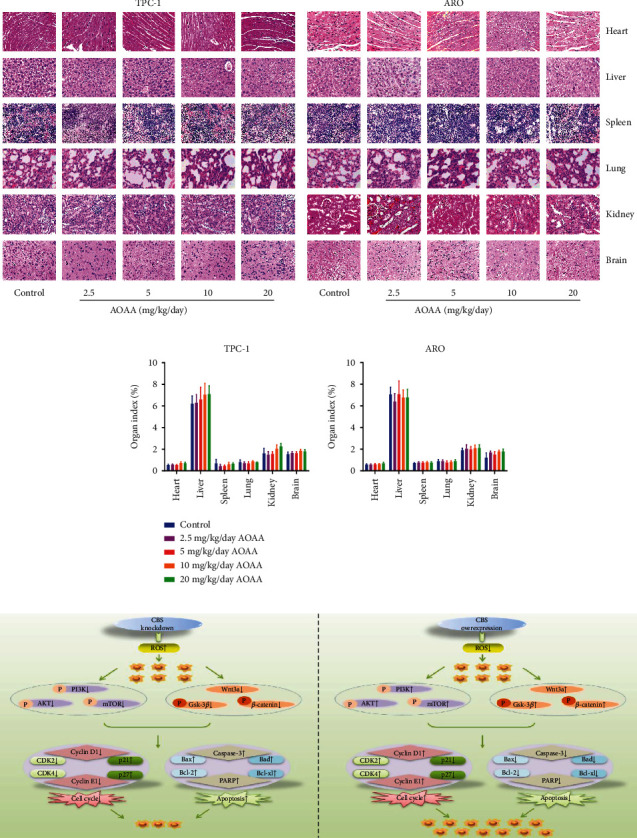
Effects of AOAA on the toxicity in nude mice. (a) Representative figures of the heart, liver, spleen, lung, kidney, and brain in nude mice. (b) The organ index was calculated. (c) Current working model of CBS-mediated signaling pathway in the development and progression of human thyroid carcinoma cells.

AOAA is one of the most commonly used pharmacological inhibitors of CBS [44–46]. The effect of AOAA on the growth of human thyroid carcinoma cells was further determined. We found that AOAA dose-dependently suppressed proliferation, viability, migration, and invasion of thyroid carcinoma cells (Figure 8 and S5). In addition, AOAA suppressed thyroid carcinoma xenograft growth in a dose-dependent manner (Figures 9(a)–9(d) and S6a, b). AOAA dose-dependently downregulated the proliferation and MVD of human NPC xenograft tumors, but showed reverse effects on p21 expression and apoptotic index (Figures 9(e)–9(i)). However, there was no morphological difference of heart, liver, spleen, lung, kidney, and brain among groups. Moreover, no obvious difference in organ index and body weight was observed among groups (Figures 10(a) and 10(b) and S6c, d). The data together suggest that CBS inhibitor could suppress human thyroid carcinoma cell growth without significant toxicity.

## 4. Discussion

Thyroid cancer is one of the most prevalent malignancies in endocrine system, and its incidence has continuously increased during the past few decades worldwide [19]. It has been revealed that abnormal H_2_S metabolism is involved in several diseases, such as cancer [16]. Our previous study has indicated that administration of exogenous H_2_S can regulate the growth of human thyroid carcinoma cells [20]. Furthermore, a recent study has shown that the expression level of CBS in thyroid carcinoma is higher than that in benign thyroid [21]. In line with the previous study, our results indicated that CBS level was higher in thyroid carcinoma tissues than that in adjacent nontumor tissues. Moreover, CBS expression was found to be associated with T classification of thyroid carcinoma. According to these findings, it can be concluded that CBS level is high in thyroid cancer tissues and low in adjacent nontumor tissues, indicating that CBS is an important biomarker for the diagnosis and prognosis of thyroid cancer and can play a role in the development of thyroid cancer.

Many studies have shown that CBS is involved in the growth of several types of cancer, such as colon cancer [16] and liver cancer [47]. Nevertheless, the effect and mechanism of CBS on the growth of human thyroid carcinoma cells remain unknown. The human thyroid carcinoma cell lines TPC-1 and ARO are used to determine the therapeutic effects of different agents [20, 36]. In the current study, these two cells were adopted to detect the mechanism of action of CBS on the growth of thyroid carcinoma both *in vitro* and *in vivo.* It has been shown that overexpression of CBS enhances migration, invasion, anchorage-independent growth, and tumorigenicity of NCM356 colonic epithelial cells [48]. Another study indicates that shRNA-mediated downregulation of CBS inhibits the proliferative, migrative, and invasive activities of HCT116 colon cancer cells *in vitro* and suppresses colon cancer growth and tumor angiogenesis *in vivo* [16]. Similarly, our results suggested that CBS overexpression upregulated the viability, proliferation, migration, and invasion capabilities of TPC-1 and ARO cells. In addition, overexpression of CBS increased the population of cells in G2 phase and decreased the percentage of cells in S phase. However, CBS knockdown exhibited completely opposite effects. Collectively, these results indicate that CBS is involved in the regulation of the growth, migration, invasion, and cell cycle of human thyroid carcinoma cells.

Apoptosis is responsible for the development and maintenance of tissue homeostasis in multicellular organisms [49]. There are two main pathways of apoptosis in mammals: the mitochondria-mediated intrinsic pathway and death receptor-mediated extrinsic pathway [50]. Caspases could be activated in response to various proapoptotic stimuli and PARP is proteolytically cleaved by caspase-3, resulting in the occurrence of apoptosis [51]. Treatment with 400 *μ*mol/l NaHS (an H_2_S donor) for 24 h markedly reduces apoptosis, as indicated by the decreased levels of cleaved caspase-3 and Bax and increased Bcl-2 expression in human esophageal carcinoma EC109 cells [52]. Another study indicates that knockdown of CBS can induce the increase in the Bax/Bcl-2 ratio, activation of caspase-3 and PARP in human hepatoma SMMC-7721 cells [47]. Similarly, our data suggested that CBS overexpression reduced both Bax/Bcl-2 and Bad/Bcl-xl ratios, as well as the expressions of cleaved caspase-3 and cleaved PARP, whereas CBS knockdown exhibited reverse effects. Thus, CBS can modulate mitochondria-dependent apoptosis in human thyroid carcinoma cells.

Considering different ROS levels could cause diverse biological responses, the modulation of intracellular ROS levels plays a key role in cellular homeostasis [37]. Low to moderate levels of ROS contribute to the initiation and progression of cancer either by promoting the mutation of genomic DNA or acting as signaling molecules. In contrast, high levels of ROS could induce cellular damage and promote cancer cell death [37, 39]. The PI3K/AKT/mTOR pathway is frequently dysregulated in cancer, and ROS can act as upstream regulators of the pathway [20, 53]. In addition, many studies have revealed that Wnt/*β*-catenin pathway can be regulated by ROS in cancer cells [54, 55]. Our data suggested that CBS overexpression decreased the ROS levels, and CBS knockdown promoted ROS generation in human thyroid carcinoma cells. There are many factors that may mediate the effects of CBS on ROS levels, such as H_2_S, homocysteine levels, and glutathione biosynthesis, which need to be further investigated. Moreover, both of these pathways are activated in several types of cancer, such as ovarian cancer [56], acute myeloid leukemia [57], and Ewing's sarcoma [58], suggesting that these two pathways may play synergistic effects in the development of cancer. We found that CBS overexpression increased the expressions of p-PI3K, p-AKT, p-mTOR, Wnt3a, p-*β*-catenin, and p-Gsk-3*β*, whereas CBS knockdown reduced the levels of these proteins. Our data together indicate that CBS modulates ROS-mediated PI3K/AKT/mTOR and Wnt/*β*-Catenin pathways in human thyroid carcinoma cells.

Recent studies have indicated that TPC-1 and ARO cells can be successfully used to establish subcutaneous xenograft tumors [20, 36]. We then determined the effects of CBS on the growth of thyroid carcinoma xenograft tumors. CBS overexpression significantly enhanced the growth of thyroid carcinoma xenograft tumors, while CBS knockdown dramatically decreased tumor growth. Ki67, a cell cycle-related protein, is a key marker in detecting the proliferation of cancer cells [23, 26, 59]. CD31 is a crucial biomarker for vascular endothelial cells, and its density has been regarded as the tumor MVD [29]. p21, a member of cyclin-dependent kinase inhibitors, plays a critical role in cell cycle arrest in many types of cancer [30, 60]. Cleaved caspase-3 has been shown to play a central role in the progression of apoptosis [35, 61]. The data suggested that CBS overexpression increased the proliferation index and MVD, but decreased the ratio of p21 positive cells and the apoptotic index. However, CBS knockdown exhibited completely reverse trends. Taken together, CBS could regulate the growth of human thyroid carcinoma xenograft tumors through the mediation of angiogenesis, cell cycle, and apoptosis.

AOAA, a CBS inhibitor, could suppress the development of different types of cancer. It has been revealed that inhibition of CBS by AOAA decreases the endogenous H_2_S levels, promotes mitochondria-mediated apoptosis, and inhibits the transcriptional activity of nuclear factor-kappa B in chronic myeloid leukemia-derived K562 cells [44]. In colon cancer cells, AOAA induces the upregulation of E-cadherin and Zonula occludens-1 and downregulation of fibronectin expression, indicating that AOAA could produce a pharmacological induction of mesenchymal-epithelial transition [45]. Another study indicates that CBS is overexpressed in colorectal cancer tissues, and AOAA could sensitize colon cancer cells to oxaliplatin by increasing intrinsic apoptosis [46]. In addition, combination of AOAA with 3,3^∗^-diindolylmethane synergistically inhibits proliferation and migration, but increases apoptosis in gastric cancer cells [62]. In according to previous findings, our data suggested that AOAA dose-dependently inhibited the viability, proliferation, migration, and invasion of thyroid carcinoma cells. Moreover, no obvious difference was found in body weight, relative organ weight, and morphologies of heart, liver, spleen, lung, kidney, and brain among groups, suggesting no obvious systemic toxicity. Therefore, AOAA can be used to inhibit thyroid carcinoma cell growth without significant toxicity.

In conclusion, our results demonstrate that the expression level of CBS in human thyroid carcinoma tissues is higher than that in adjacent nontumor tissues. We found that CBS can mediate the cell cycle, proliferation, migration, and invasion of human thyroid carcinoma cells via ROS-mediated PI3K/AKT/mTOR and Wnt/*β*-Catenin signaling pathways (Figure 10(c)). Considering its role in the progression of human thyroid carcinoma cells, CBS may act as a promising biomarker for the diagnosis and prognosis in thyroid carcinoma patients. In addition, CBS could be a novel therapeutic target and novel donors that inhibit the expression of CBS can be developed in the treatment of thyroid carcinoma.

## Figures and Tables

**Table 1:** Association between CBS expression and clinicopathological characteristics of patients with papillary thyroid carcinoma (*n* = 54).

Characteristics	Cases	CBS expression		*P* value
		Low	High	
*Age (years)*				0.745
≤44	23	16	7	
45-59	23	15	8	
≥60	8	4	4	
*Gender*				0.458
Male	23	15	8	
Female	31	20	11	
*Tumor size (cm)*				0.463
≤3	42	29	13	
>3	12	6	6	
*Disease grade*				0.762
I	31	26	5	
II	7	5	2	
III	16	4	12	
*T classification*				0.014
T1	28	20	8	
T2	22	13	9	
T3	2	1	1	
T4	2	0	2	
*Lymph node status*				0.436
N0	29	19	10	
N1	25	15	10	

## Data Availability

The data of the study are available from the corresponding author on reasonable request.

## Abbreviations

H_2_S: Hydrogen sulfide
L-Cys: L-cysteine
CSE: Cystathionine *γ*-lyase
CBS: Cystathionine *β*-synthase
3-MST: 3-Mercaptopyruvate sulfurtransferase
CAT: Cysteine aminotransferase
AOAA: Aminooxyacetic acid
IHC: Immunohistochemistry
FBS: Fetal bovine serum
cDNA: Complementary deoxyribonucleic acid
shRNA: Short hairpin ribonucleic acid
EdU: 5-Ethynyl-2′-deoxyuridine
PBS: Phosphate-buffered saline
PI: Propidium iodide
MR: Migration rate
TUNEL: TdT-mediated dUTP-biotin nick end labeling
ROS: Reactive oxygen species
DHE: Dihydroethidium
SOD: Superoxide dismutase
CAT: Catalase
GSH-Px: Glutathione peroxidase
CDK: Cyclin-dependent kinase
PI3K: Phosphatidylinositol 3-kinase
mTOR: Mammalian target of rapamycin
Gsk-3*β*: Glycogen synthase kinase-3 beta
CST: Cell signaling technology
Bcl-xl: B-cell lymphoma-extra large
Bcl-2: B-cell lymphoma-2
Bad: Bcl-xl/Bcl-2-associated death promoter
Bax: Bcl-2-associated X protein
PARP: Poly-ADP-ribose polymerase
V: Volume
TVDT: Tumor volume doubling time
IR: Inhibition rate
HE: Hematoxylin and eosin
CD31: Cluster of differentiation 31
MVD: Microvessel density
SEM: Standard error of the mean.

## Supplementary Materials

Supplementary Materials Figure S1: the expression levels of CSE and 3-MST in human thyroid carcinoma cell lines. (a) Western blotting analysis for the expression levels of CSE and 3-MST in Nthy-ori3-1, TT, ARO, TPC-1, and FTC-133 cells. *β*-Actin was used as the loading control. (b) The densitometry analyses of CSE and 3-MST were performed, normalized to the corresponding *β*-actin level. Data are presented as mean ± SEM of three independent experiments; ∗*P* < 0.05, ∗∗*P* < 0.01 compared with human thyroid epithelial cell line Nthy-ori3-1. Figure S2: effects of CBS on the concentration of H^2^S in human thyroid carcinoma cells. (a) The concentrations of H^2^S in cells were determined. (b) The concentrations of H^2^S in culture supernatant were detected. Data are presented as mean ± SEM of three independent experiments; ∗*P* < 0.05, ∗∗*P* < 0.01 compared with the Mock group; #*P* < 0.05, ##*P* < 0.01 compared with the sh-Scb group. Figure S3: effects of CBS on the activities of SOD, CAT, and GSH-Px in human thyroid carcinoma cells. (a) The activities of SOD, CAT, and GSH-Px were determined. Data are presented as mean ± SEM of three independent experiments; ∗∗*P* < 0.01 compared with the Mock group; ##*P* < 0.01 compared with the sh-Scb group. Figure S4: effects of CBS on the PI, MVD, cell cycle, and apoptosis of human thyroid carcinoma xenografts. (a) Representative photographs of HE staining in TPC-1 and ARO xenograft tumors (original magnification 400 ×). (b) Representative photographs of Ki67 staining in TPC-1 and ARO xenograft tumors (original magnification 400 ×) and the PI were calculated. (c) Representative photographs of CD31 staining in TPC-1 and ARO xenograft tumors (original magnification 400 ×) and the MVD was calculated. (d) Representative photographs of p21 staining in TPC-153 and ARO xenograft tumors (original magnification 400 ×) and the p21 positive cells were calculated. (e) Representative photographs of cleaved caspase-3 staining in TPC-1 and ARO xenograft tumors (original magnification 400 ×) and the apoptotic index were calculated. Values are presented as mean ± SEM (*n* = 6); ∗∗*P* < 0.01 compared with the Mock group; ##*P* < 0.01 compared with the sh-Scb group. Figure S5: effects of AOAA on the migration and invasion of human thyroid carcinoma cells. (a) The effect of AOAA on cell migration was measured by wound healing assay; original magnification 100×. (b) The migration rates of TPC-1and ARO cells were calculated. (c) Soft agar assay was performed to examine the anchorage-independent survival of cells; original magnification 100×. (d) The number of colonies was calculated. Data are presented as mean ± SEM of three independent experiments; ∗*P* < 0.05, ∗∗*P* < 0.01 compared with the control group. Figure S6: effects of AOAA on DT/DC and TVDT of human thyroid carcinoma xenografts and body weight of nude mice. (a) DT/DC was calculated. (b) TVDT was calculated. (c) The body weight change curve of each group during the experiment. (d) The body weight of each group on the first day (day 0) and the last day (day 28). Values are presented as mean ± SEM (*n* = 6); ∗∗*P* < 0.01 compared with the control group.
