# Supplementary figures and images for: Cystathionine β-Synthase Regulates the Proliferation, Migration, and Invasion of Thyroid Carcinoma Cells
Source: Oxid Med Cell Longev. 2022 Jun 27;2022:8678363. doi: 10.1155/2022/8678363 (PMC9252770; doi:10.1155/2022/8678363)

Figure S1

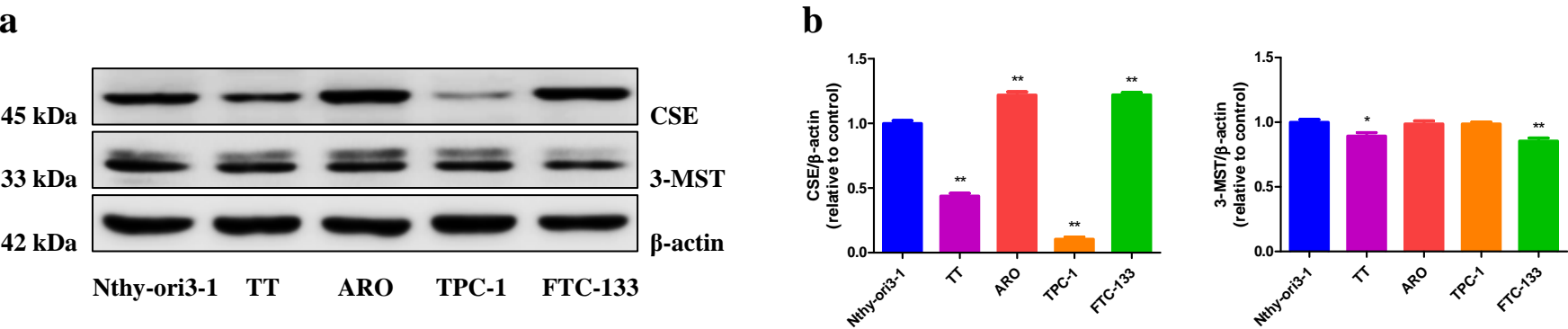

Figure S2

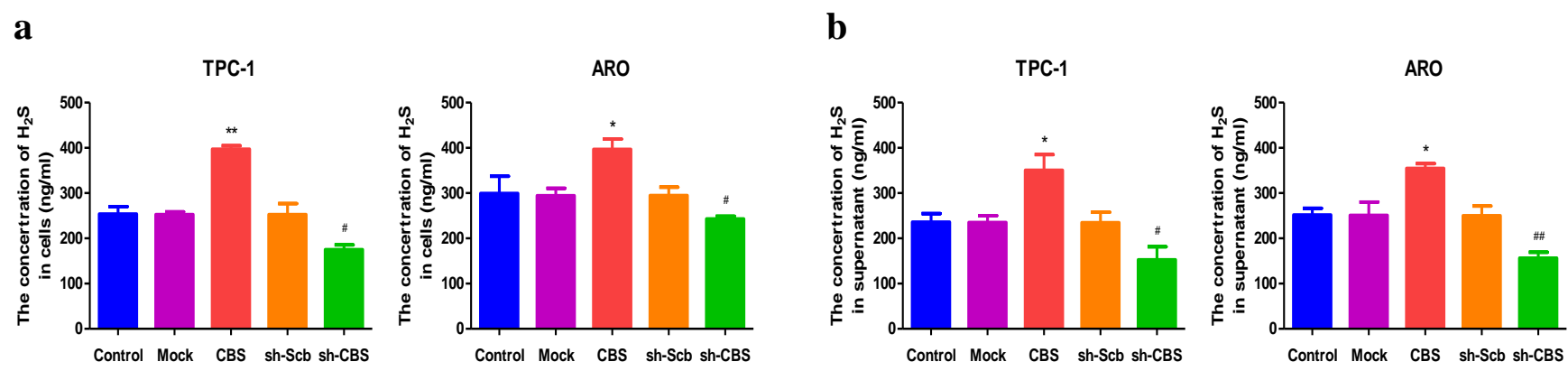

Figure S3

a

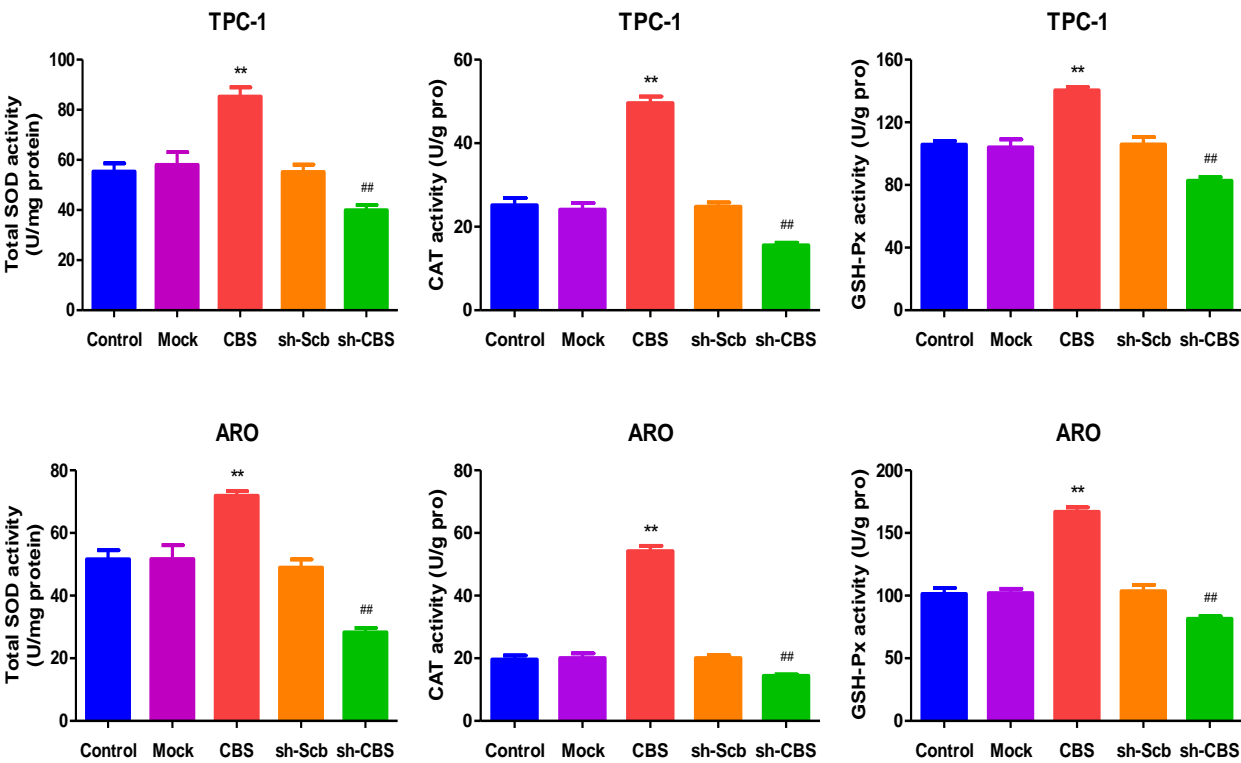

Figure S4

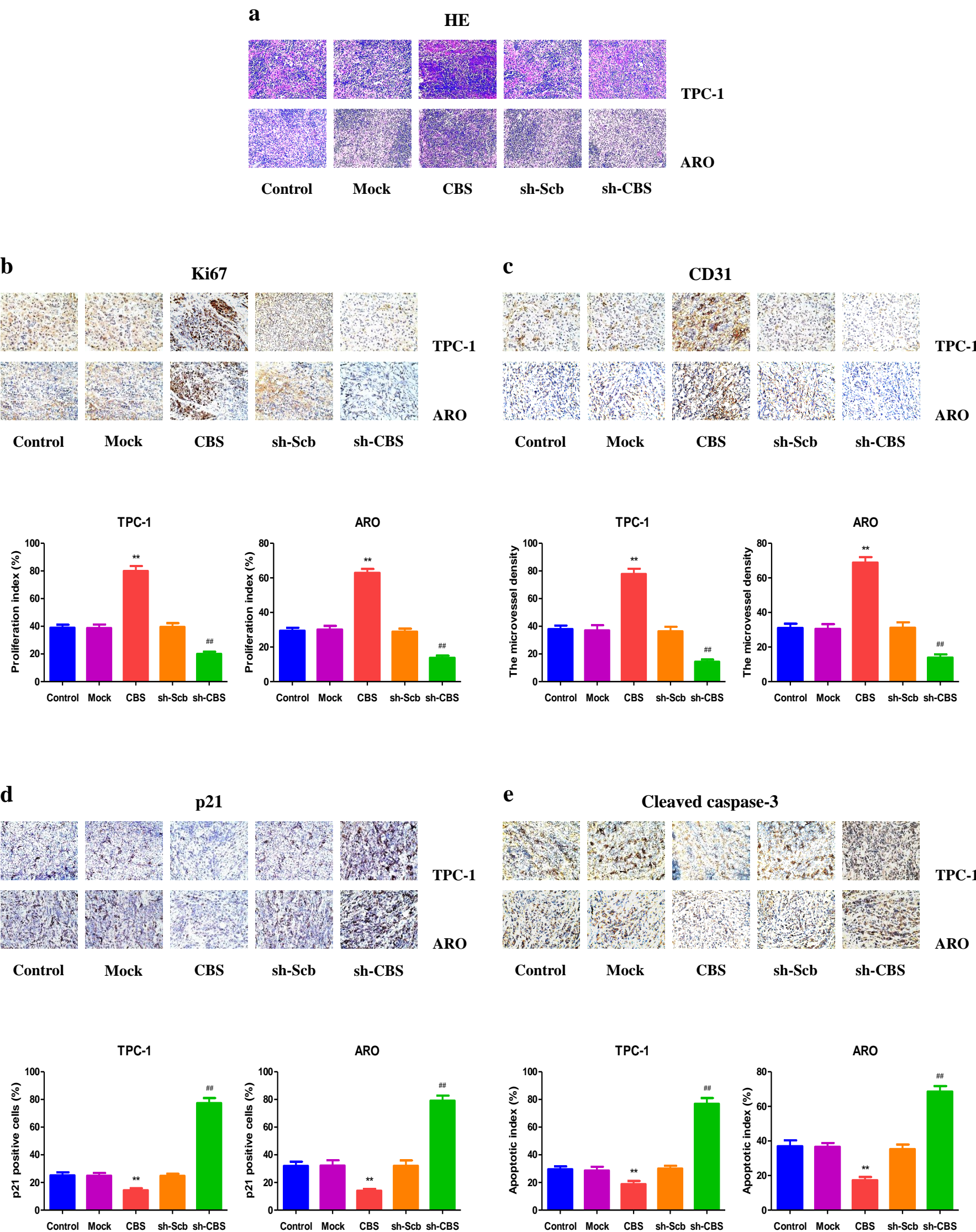

Figure S5

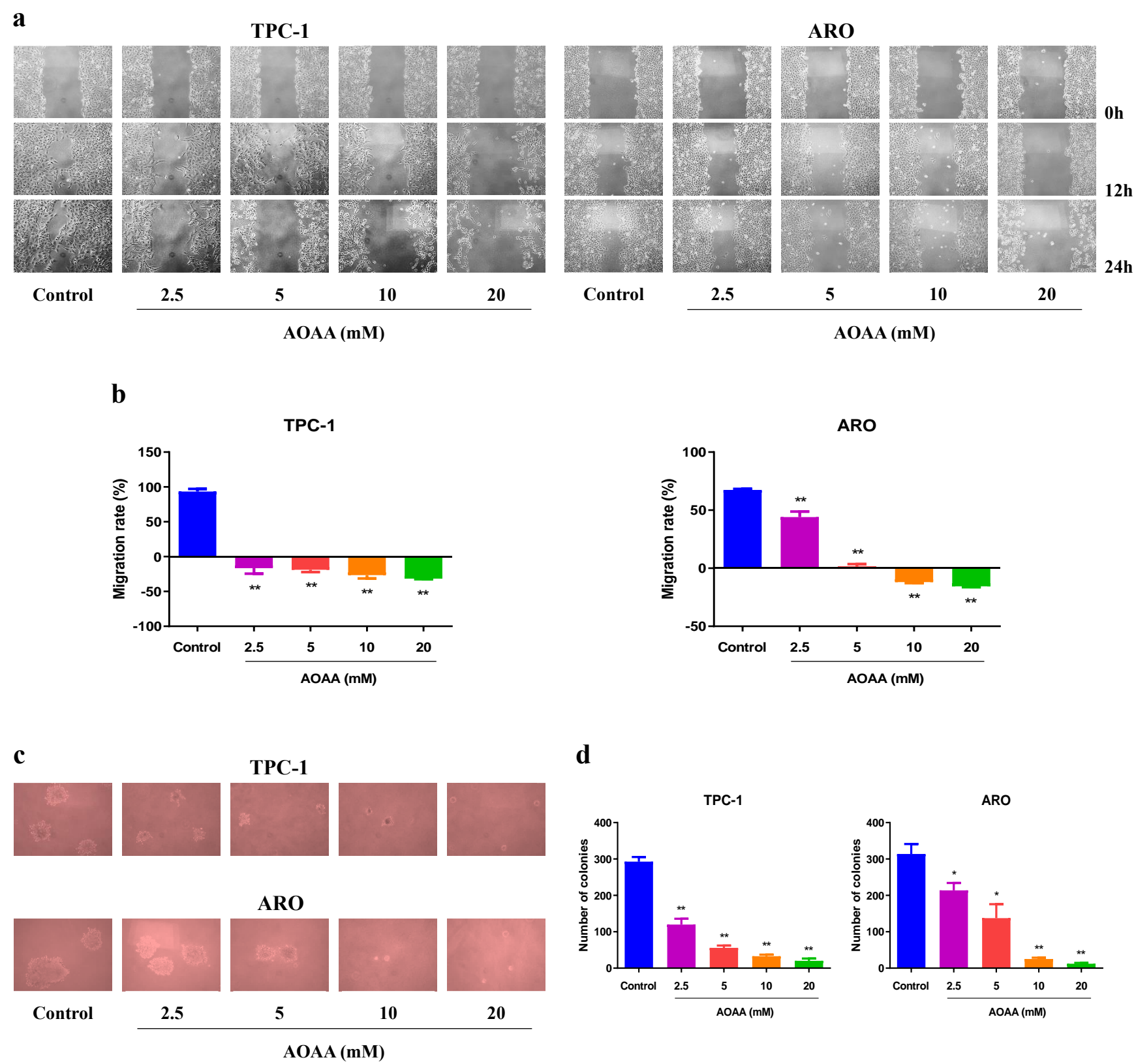

Figure S6

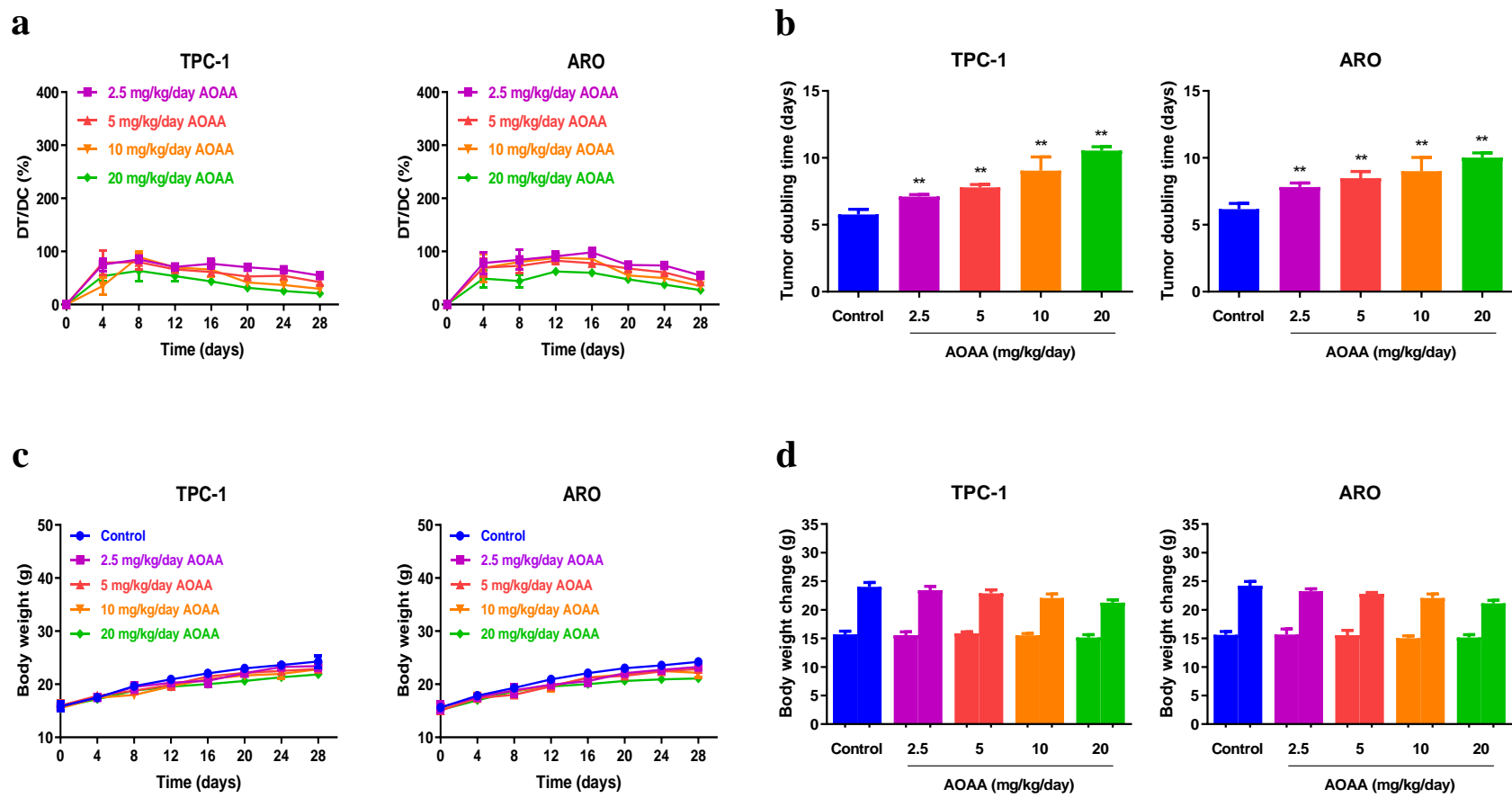

Supplement: Supplementary Materials — Figure S1: the expression levels of CSE and 3-MST in human thyroid carcinoma cell lines. (a) Western blotting analysis for the expression levels of CSE and 3-MST in Nthy-ori3-1, TT, ARO, TPC-1, and FTC-133 cells. β-Actin was used as the loading control. (b) The densitometry analyses of CSE and 3-MST were performed, normalized to the corresponding β-actin level. Data are presented as mean ± SEM of three independent experiments; ∗P < 0.05, ∗∗P < 0.01 compared with human thyroid epithelial cell line Nthy-ori3-1. Figure S2: effects of CBS on the concentration of H2S in human thyroid carcinoma cells. (a) The concentrations of H2S in cells were determined. (b) The concentrations of H2S in culture supernatant were detected. Data are presented as mean ± SEM of three independent experiments; ∗P < 0.05, ∗∗P < 0.01 compared with the Mock group; #P < 0.05, ##P < 0.01 compared with the sh-Scb group. Figure S3: effects of CBS on the activities of SOD, CAT, and GSH-Px in human thyroid carcinoma cells. (a) The activities of SOD, CAT, and GSH-Px were determined. Data are presented as mean ± SEM of three independent experiments; ∗∗P < 0.01 compared with the Mock group; ##P < 0.01 compared with the sh-Scb group. Figure S4: effects of CBS on the PI, MVD, cell cycle, and apoptosis of human thyroid carcinoma xenografts. (a) Representative photographs of HE staining in TPC-1 and ARO xenograft tumors (original magnification 400 ×). (b) Representative photographs of Ki67 staining in TPC-1 and ARO xenograft tumors (original magnification 400 ×) and the PI were calculated. (c) Representative photographs of CD31 staining in TPC-1 and ARO xenograft tumors (original magnification 400 ×) and the MVD was calculated. (d) Representative photographs of p21 staining in TPC-153 and ARO xenograft tumors (original magnification 400 ×) and the p21 positive cells were calculated. (e) Representative photographs of cleaved caspase-3 staining in TPC-1 and ARO xenograft tumors (original magnification 400 ×) an [file 8678363.f1.pdf]
